# Indirect Estimation of the Comparative Treatment Effect in Pharmacogenomic Subgroups

**DOI:** 10.1371/journal.pone.0072256

**Published:** 2013-08-27

**Authors:** Michael J. Sorich, Michael Coory, Brita A. K. Pekarsky

**Affiliations:** 1 School of Pharmacy and Medical Sciences, University of South Australia, Adelaide, South Australia, Australia; 2 School of Medicine, Flinders University, Adelaide, South Australia, Australia; 3 Health Services Research, Murdoch Childrens Research Institute, Melbourne, Victoria, Australia; 4 Health and Lifestyle Laboratory, Baker IDI Heart and Diabetes Institute, Melbourne, Victoria, Australia; Universite de Montreal, Canada

## Abstract

Evidence of clinical utility is a key issue in translating pharmacogenomics into clinical practice. Appropriately designed randomized controlled trials generally provide the most robust evidence of the clinical utility, but often only data from a pharmacogenomic association study are available. This paper details a method for reframing the results of pharmacogenomic association studies in terms of the comparative treatment effect for a pharmacogenomic subgroup to provide greater insight into the likely clinical utility of a pharmacogenomic marker, its’ likely cost effectiveness, and the value of undertaking the further (often expensive) research required for translation into clinical practice. The method is based on the law of total probability, which relates marginal and conditional probability. It takes as inputs: the prevalence of the pharmacogenomic marker in the patient group of interest, prognostic effect of the pharmacogenomic marker based on observational association studies, and the unstratified comparative treatment effect based on one or more conventional randomized controlled trials. The critical assumption is that of exchangeability across the included studies. The method is demonstrated using a case study of cytochrome P450 (CYP) 2C19 genotype and the anti-platelet agent clopidogrel. Indirect subgroup analysis provided insight into relationship between the clinical utility of genotyping CYP2C19 and the risk ratio of cardiovascular outcomes between CYP2C19 genotypes for individuals using clopidogrel. In this case study the indirect and direct estimates of the treatment effect for the cytochrome P450 2C19 subgroups were similar. In general, however, indirect estimates are likely to have substantially greater risk of bias than an equivalent direct estimate.

## Introduction

An important element of pharmacogenomics is the use of genomic information (genetic variation and gene expression) to enable stratified or personalised medicine. In particular, there is great interest in use of pharmacogenomic markers to guide medical decisions regarding the best choice of therapy. Evidence of clinical utility for a given marker is a key issue in translating pharmacogenomics into clinical practice [1] and the extent to which comparative treatment effect differs between subgroups defined by the marker is an important component of assessing clinical utility. We define clinical utility here as the improvement in clinical outcomes (i.e., evidence of health gain) resulting from use of a pharmacogenomic test [2]. We exclude from the concept of clinical utility the dimension of cost effectiveness (value for money) of the pharmacogenomic marker in producing the health gain, although we discuss the application of the method to pharmacoeconomic modelling.

Appropriately designed randomised controlled trials (RCTs) can provide robust evidence of the relationship between treatment effect and pharmacogenomic marker status [3]. However, RCT evidence is not always available. Association studies of pharmacogenomic markers are much more common but the results of such studies are less useful for providing insight of the clinical utility. Pharmacogenomic association studies are typically observational cohort or case-control studies which assess the association between a pharmacogenomic marker and clinical/surrogate outcomes for a specific patient population on a specific treatment. Typically the results of a pharmacogenomic association study will highlight that individuals with one value for the marker are at higher risk of an event when using a specific drug, compared to individuals who have a different value for the marker. However, this is generally insufficient to inform whether the pharmacogenomic marker identifies subgroups with clinically important and statistically significant differences in comparative treatment effects.

This paper describes the mathematical basis and assumptions of a method for indirectly estimating comparative treatment effect for subgroups defined by a pharmacogenomic marker based on data commonly available for the patient population of interest: pharmacogenomic association studies, the prevalence of the marker, and treatment effect in the unstratified population. A case study for the use of this method is presented, based on the cytochrome P450 (CYP2C19) genotype subgroup analysis of the RCT comparing ticagrelor and clopidogrel for the prevention of cardiovascular (CV) events for individuals with acute coronary syndrome (ACS). Evidence generated using this approach is not a substitute for direct evidence from an RCT; however, combined with a sensitivity analysis, this indirect method can provide insight into whether the pharmacogenomic marker is likely to have clinical utility and/or be cost-effective, and hence the value of undertaking further research.

## Methods

The general approach developed below is to construct a hypothetical trial that embodies the known characteristics of the treatment and pharmacogenomic marker – the overall treatment effect unstratified by the marker, the marker effect in each study arm, and the distribution of the marker. The comparative treatment effect for the marker subgroups is estimated by demonstrating that only specific values of the treatment effect for the subgroups will be consistent with the set of treatment and marker characteristics specified.

If an appropriately designed RCT, comparing treatments α and β, were available in which the pharmacogenomic marker status for participants is known, a subgroup analysis may be undertaken on the basis of the marker. For simplicity it is assumed here that the marker only has two values (A and A′; e.g. corresponding to positive/negative, high/low, mutated/wildtype, carriage of allele/no carriage of allele) and that the outcome of interest is a binary event (e) that has a probability (P) of occurring over a specified time period. For each marker subgroup the risk ratio (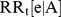 and 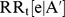) for the comparative treatment effect may be directly estimated from such an RCT. As indicated by equation 1, the information derived from such a trial would be sufficient to determine the choice of therapy (*α* or *β*) for each subgroup that will minimize the risk of the event. However, such trials are not always available. Therefore, the specific goal of the analysis presented in this paper is to indirectly estimate 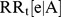 and 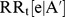.
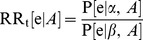
(1)


A common form of evidence for a pharmacogenomic marker is an association study. Data from an association study (or meta-analysis of association studies) provides an estimate of the risk ratio of an outcome between individuals with different values of the marker for individuals using treatment α (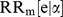: equation 2). A similar estimate may be available for individuals using an alternative treatment β (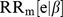).
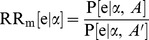
(2)


With this information, a prescriber can advise a patient of his or her prognosis given the use of either drug. However, this information is insufficient to advise the patient as to the optimum choice of therapy; that which minimizes P[e]. Specifically, if 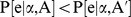 it does not follow that patients with the marker value A′ should not be treated with therapy α, which could still be more effective compared to alternative treatment options (e.g. β).

In addition to estimates of 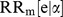 and 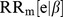 from association studies it is assumed that an estimate of the treatment effect is available from a conventional RCT (or meta-analysis of RCTs), in which the cohort is not stratified for the marker of interest (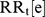). Alternatively, 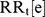 may be based on an indirect treatment comparison of RCTs with a common comparator although this may lead to an increased the risk of bias [4], [5]. Third, it is assumed that data is available on the prevalence of the marker in patients who have the condition that will be treated with α or β. This information is generally available from the association studies but may also be sourced elsewhere. It is assumed that the prevalence of the marker is balanced between arms of the hypothetical trial.

The probability of the clinical outcome in the unstratified cohort is estimated to be the weighted average of the probability of the clinical outcome in the pharmacogenomic subgroups, using the law of total probability, which relates marginal probability and conditional probability (equation 3).
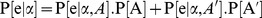
(3)


Combining equations 2 and 3 leads to the following formulas for indirectly estimating risk of the event in the pharmacogenomic subgroups (A and A′) for treatment α. Calculation of the risk of the event in the pharmacogenomic subgroups for treatment β may be similarly undertaken.
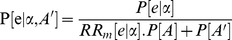


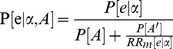



Subsequently, using the relationship described in equation 1 the comparative treatment effect for the subgroups defined by the pharmacogenomic marker may be indirectly estimated.
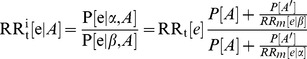


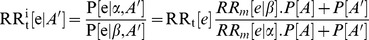



Credible intervals (analogous to confidence intervals) for pharmacogenomics subgroup treatment effects and the statistical inference on the difference between subgroup treatment effects may be estimated using Monte Carlo simulation. This approach essentially estimates the uncertainty of the output (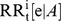 and 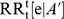) based on the collective uncertainty of the inputs (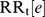,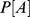,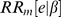, and 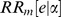). Thus, information on the distribution of the above parameters (e.g. based on the 95% confidence interval) would need to be available. Typically risk ratio estimates are represented by a lognormal distribution and probabilities by a beta distribution [6]. Monte Carlo simulation involves randomly drawn values from the distributions of the input variables and the calculation of the output variable. This process is repeated a large number of times (e.g. 10,000) producing the distribution of the output variable. Assessment of whether the difference between subgroups is statistically significant (statistical test of interaction) may also be estimated [7]. However, care must be taken in interpreting the statistical significance due to the risk of bias inherent in the indirect estimation.

The key assumption of the method is exchangeability of the studies (association studies, RCT). Specifically, the study populations should not differ on any modifiers of the prognostic effect of the marker or for any modifiers of the predictive effect of the marker. We introduce the label “marker-modifiers” to encompass both prognostic and predictive modifiers. Candidate marker-modifiers include patient factors (age, sex, severity of index condition, co-existing disease, ethnicity), study factors (length of follow-up, intensity of surveillance) and treatment factors (concomitant medications, surgery, or dose and duration of the index treatment).

Note that these factors could have different distributions in the included studies without invalidating the assumption of exchangeability. It is only when differences in these factors affects outcome in groups defined by the marker (i.e., only when a factor is a marker-modifier) that the assumption of exchangeability does not hold. In general, the greater the degree to which the assumption of exchangeability does not hold, the greater the expected risk of bias for comparative treatment effect estimates of the pharmacogenomic subgroups. The assumption of exchangeability in this context is analogous to the assumption of exchangeability (sometimes called “similarity”) of RCTs in an indirect treatment comparison; or more broadly of exchangeability for RCTs, non-randomised studies and direct head-to-head studies in a network meta-analysis. The variables (if any) that can modify the pharmacogenomic association study effect size and the direction of the modification will tend to be specific to the marker and drug in question and hence it is not possible to make a generic statement of how factors will affect exchangeability. The marker prevalence is unlikely to be an issue with respect to exchangeability unless there are substantial differences in marker prevalence between studies and marker prevalence is believed to modify the marker effect.

It is also assumed that the contributing studies are methodologically sound and their results are not subject to bias. In general, the greater the risk of bias in the contributing studies, the greater the expected risk of bias for comparative treatment effect estimates of the pharmacogenomic subgroups. The inputs and assumptions of the approach are summarized in Table 1.

**Table 1:** Required inputs and assumptions of the indirect estimation approach.

Required Inputs	Assumptions
Prevalence of the pharmacogenomic marker in the patient group of interest	Available studies can be generalised to the patient group of interest
A measure of the strength of association between pharmacogenomicmarker and prognosis in the patient group of interest using the treatmentsof interest	The included studies are exchangeable; that is they do not differ significantly on patient, treatment, or study characteristics that are marker-effect modifiers
A measure of the unstratified comparative treatment effect of thetreatments of interest in the patient group of interest	The included studies are methodologically sound and their results are not subject to bias

## Case Study

A contemporary example of a pharmacogenomic marker is the use of CYP2C19 genotype to guide use of the anti-platelet agent clopidogrel. CYP2C19 loss-of-function (LoF) alleles are associated with decreased effect of clopigogrel leading to increased risk of adverse CV events [8]–[10]. An example of a treatment decision that may be influenced by CYP2C19 genotype is the choice of clopidogrel or ticagrelor following ACS. This example is particularly pertinent as a direct pharmacogenomic subgroup analysis has been published which enables a simple comparison of direct and indirect approaches [11].

In the PLATO RCT the hazard ratio for CV events was reported to be 0.84 (95% CI; 0.77 to 0.92) for ticagrelor compared to clopidogrel [12]. Due to the relatively low CV event rate in this scenario the hazard ratio is a good approximation of 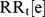 (a risk ratio). Meta-analyses of association studies have indicated significant statistical heterogeneity and report summary estimates of the risk ratio of CV outcomes for individuals using clopidogrel carrying a CYP2C19 LoF allele (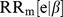) ranging from approximately 1.10 to 1.60 [8], [9], [13]. It was assumed that there was no association between CYP2C19 genotype and CV outcomes for individuals that are not taking clopidogrel (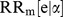 = 1) for three reasons: there is no known biological/pharmacological basis for CYP2C19 genotype to influence CV outcomes in the absence of clopidogrel therapy, the evidence from pharmacokinetic and pharmacodynamics studies indicates no effect, and association studies have not indicated any significant difference in CV risk in the absence of clopidogrel [11], [14]–[17]. The probability of carriage of a CYP2C19 LoF allele (P[A]) in a predominantly Caucasian population was estimated to be 28.0% [11], [14], [18], [19].

**Figure 1:**
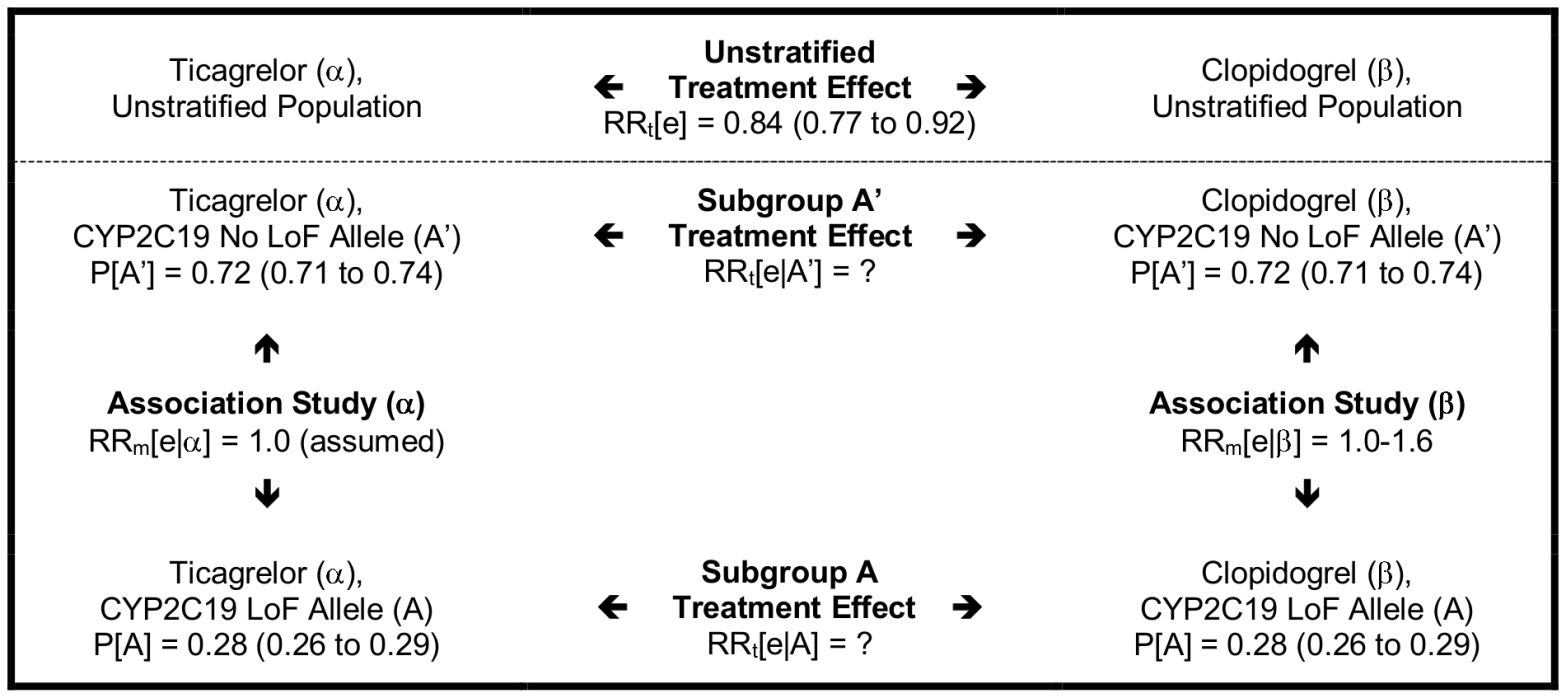
Relationships between subgroup treatment effects, association study results and unstratified RCT study results. CYP2C19 genotype and clopidogrel is used here as an example to illustrate the groups of individuals (based on treatment and pharmacogenomics marker status) involved in the indirect subgroup analysis and the relationships between the groups (both known and unknown). Values in the brackets represent the 95% confidence intervals for the estimate. CYP2C19: cytochrome P450 2C19, LoF: loss-of-function.



The relationship between the multiple sources of information used in the indirect subgroup analysis is summarised in Figure 1. A spreadsheet implementing the indirect subgroup analysis for the case study provides an example of how the calculations may be undertaken (see File S1). As an example of using the formulas derived here, the treatment effect of ticagrelor compared to clopidogrel in the subgroup that does not have a CYP2C19 LoF allele (i.e. good responders to clopidogrel) is estimated below for a relatively high value of the association between CYP2C19 genotype and CV outcomes with use of clopidogrel (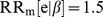).

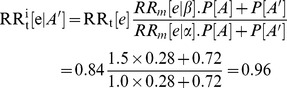



**Figure 2:**
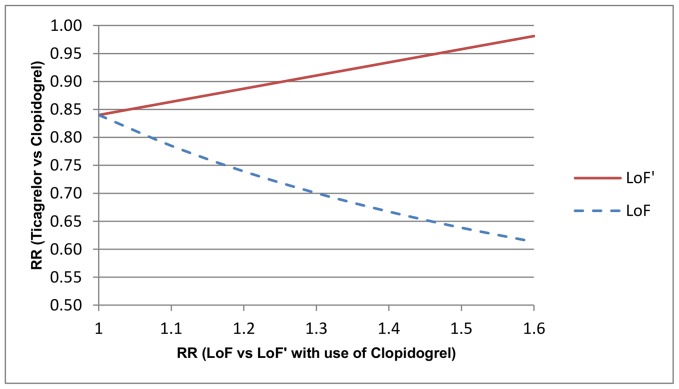
One way deterministic sensitivity analysis for indirect estimates of treatment effect. The indirect estimates of the treatment effect (relative risk for comparison of ticagrelor and clopidogrel) for subgroups based on cytochrome P450 2C19 (CYP2C19) genotype are displayed as a function of the size of the association study estimate. LoF = subgroup with a CYP2C19 loss-of-function allele, LoF′ = subgroup without a CYP2C19 loss-of-function allele.

Figure 2 displays a deterministic sensitivity analysis of the indirect estimates of treatment effect (ticagrelor compared to clopidogrel) for CYP2C19 genotypes as a function of the association study results (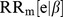). This figure helps translate an association study result into a comparative treatment effect for each pharmacogenomic subgroup and hence provides insight into whether screening for the pharmacogenomic marker is likely to result in improved patient outcomes (i.e. clinical utility). The subgroup comparative treatment effect estimates may also form the basis of formal cost-effectiveness modeling. In addition, a probabilistic sensitivity analysis was undertaken utilising Monte Carlo simulation. Using 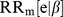 ( = 1.18 [95% CI; 1.09 to 1.28]) from a recent meta-analysis of association studies [9], 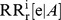 and 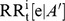 were estimated to be 0.75 (95% CI; 0.67 to 0.83) and 0.88 (95% CI; 0.81 to 0.97), respectively. This compares reasonably well to the direct estimates based on the genetic substudy of the PLATO RCT: 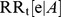 = 0·77 (95% CI; 0·60 to 0·99) and 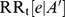 = 0·86 (95% CI; 0·74 to 1·01) [11].

## Discussion

This paper describes the mathematical basis and key assumption (i.e., exchangeability) underlying a method for indirect estimation of the comparative treatment effect in a pharmacogenomic subgroup. The method is useful for estimating the potential clinical utility of a pharmacogenomic marker, given the available data (e.g. [20], [21]); especially when sensitivity analyses are conducted around the inputs. It would be straight forward to incorporate the method into a network meta-analysis that included both direct and indirect evidence for the unstratified treatment effect [22]. Also, the method is a useful addition to the toolbox of methods available to assist in assessing the possible cost-effectiveness of a pharmacogenomic marker (e.g. [23]–[25]). In that context it provides a clear mathematical structure for synthesising the available evidence and transparency about the underlying assumption (i.e., exchangeability). It lends itself naturally to either deterministic or probabilistic sensitivity analysis [6].

The major caveats of the method relate to the assumption of exchangeability. Specifically, study populations must be similar with regard to any marker-effect modifiers (moderators of either the treatment-independent [prognostic] effect of the marker or the treatment-marker interaction effect). This is analogous to the assumption for indirect treatment comparisons where exchangeability is with respect to moderators of treatment effect. As with indirect comparisons of treatment effects it is also prudent that indirect pharmacogenomics subgroup analyses should include a detailed narrative comparison of differences in patient, study or treatment factors across the included studies. However, such differences do not necessarily mean that the assumption of exchangeability is invalidated. Evidence that factors with different distributions across included studies are also marker-effect modifiers would be required. This could be evidence from studies external to the indirect comparison or knowledge of the pathophysiology of the disease [26].

One example of violation of the assumption of exchangeability could be length of follow-up if the proportional hazards assumption does not hold [27]. If the RCT has median follow-up for 3 month, and the association study has follow-up for 1 year this may bias the subgroup treatment effect estimated if the relative risk of the association study attenuates with longer follow-up (e.g. RR would have been 0.6 rather than 0.8 if length of follow up had been 3 months instead of 1 year). The dose of the drug may modify the effect of the pharmacogenomic marker (e.g. irinotecan dose modifies the effect of UDP glucuronosyltransferase 1A1 genotype on irinotecan toxicity but not tumor response [28], [29]) and thus if the RCT and association studies have different irinotecan doses this will bias the subgroup treatment toxicity estimates. Pharmacogenomic marker effect may also vary between patient populations (e.g. between different subtypes or stages of the disease). Unexplained heterogeneity for pharmacogenomic marker effect is also problematic. The clopidogrel case study is a good example in which the effect of CYP2C19 genotype varies significantly between studies, but the reason for the variation is not well understood [9], [10]. Consequently, it is very difficult to be certain that the studies are sufficiently similar in terms of the (unknown) important characteristics that can modify the pharmacogenomic marker effect.

Formulas for indirect estimation of subgroup effects for a pharmacogenomic marker are based on the total law of probability and are therefore presented in terms of risk ratios (RR). There are other commonly used relative measures of treatment effect: odds ratio and hazard ratio. If the baseline event risk is small (say <10%) then these measures will be approximately equal to the risk ratio and could be substituted for them in the formulas (as is the case for the case study above where the hazard ratio was substituted for the risk ratio). Further research is required assess the best approach to indirectly estimate the subgroup treatment effects when event rates are significantly higher (e.g. advanced cancer). Additionally, the formulas presented are applicable to the common situation in which a marker with two levels (e.g. high/low, mutant/wildtype) is used to predict a dichotomous outcome (e.g. event or no event). The general principles used to derive the formulas should be generalizable to other situations (e.g. continuous/multi-level markers/outcomes) although the formulas are likely to be more complex. A simple option is to convert such data (e.g. dichotomize a continuous marker or outcome) to enable the application of the formulas presented here although it is important to be cognizant that in some cases this may result in significant loss of information.

The relationships presented here highlight the importance of understanding association between pharmacogenomic groups and events in the presence and absence of the drug in question (i.e. both 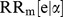 and 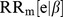). Such information is required to estimate whether the marker is prognostic and/or a predictive modifier. In the absence of both of these values, it is still possible to undertake a sensitivity and scenario analysis based on plausible assumptions to better understand the value of undertaking further research. Plausible scenarios may include that the marker is not associated with the outcome in the absence of a specific drug (e.g. CYP2C19 genotype is not associated with CV events when clopidogrel is not being used), or that the association is of similar size to that estimated in the presence of the drug (indicating a marker that is prognostic rather than a modifier of a specific treatment effect).

In the case study presented here, a deterministic sensitivity analysis facilitated insight into clinical utility by reframing the association study results in terms of plausible subgroup treatment effects. Given that there is still substantial uncertainty and risk-of-bias with respect to the association study results for clopidogrel and *CYP2C19* genotype, the sensitivity analysis (Figure 2) enables the reader to readily appreciate how the indirect estimate would be affected if the association effect size differs from the value used. In addition, Monte Carlo simulation was used to estimate the distribution of the subgroup treatment effects. The direct and indirect estimates of the subgroup treatment effects agreed reasonably well in the case study. However, an important direction of future research will be to undertake a more comprehensive assessment of inconsistency between direct and indirect approaches, as has been recently undertaken for indirect treatment comparisons [4].

It is valuable to have insight into the expected clinical utility of a proposed pharmacogenomic marker as early as possible in order to assess the likely value of undertaking an RCT designed to produce higher quality evidence of the clinical utility [30], [31]. Techniques such as value of information analysis may be utilised to explicitly and quantitatively estimate the value of undertaking further research [23], [32]. In the absence of RCT data on the value of utilising a marker the indirect approach described here allows reframing of association study results in terms of a treatment effect in subgroups defined by a pharmacogenomic marker. This reframe can allow greater insight of clinical utility, in particular whether testing for the marker is likely to result in improved clinical decisions regarding treatment selection.

## Supporting Information

File S1 Ms Excel spreadsheet with example calculations based on the clopidogrel pharmacogenetics case study.
(XLSX)
